# Assessment of Both Personal and Professional Aspects to Measure Job Satisfaction Levels among Garment Workers: Empirical Evidence from a Developing Country

**DOI:** 10.3390/ijerph192416868

**Published:** 2022-12-15

**Authors:** Deli Yuan, Md. Abu Issa Gazi, Md. Alinoor Rahman

**Affiliations:** 1 School of Management, Jiujiang University, Jiujiang 332005, China; 2 Department of Management, Islamic University, Kushtia 7003, Bangladesh

**Keywords:** job satisfaction, personal aspects, professional aspects, garments, workers

## Abstract

The main objective of the present study is to assess the role of professional and personal aspects in determining job satisfaction of garment industry workers in Bangladesh as a developing country. The present study is conducted on 350 workers from 25 garment factories in Dhaka, Narayanganj, and Gazipur, three districts of Bangladesh where the garment industry dominates. The study location and sample size were determined based on the random sampling method. All the participating workers were interviewed in the light of a predetermined questionnaire. Statistical Package for Social Science-SPSS software, version 24.0 was used for data analysis. Brayfield–Rothe Job Satisfaction Measurement Scale (JSMS) and Questionnaire for Measuring Satisfaction with professional aspects (QMSPA) has been used in this study. The results of the current study showed that, among personal aspects, only age significantly impacted the respondents’ overall job satisfaction. The respondents’ overall job satisfaction was also significantly influenced by all professional factors. Between the personal and professional facets, the importance of professional factors as determinants of job satisfaction is greater than that of personal factors. The results of the present study suggest that since professional aspects are the main components of job satisfaction, it is necessary to ensure the proper presence of these components. Factors such as pay, job security, and adequate and appropriate amount are indicators of job satisfaction. All parties involved in the garments industry such as employer–worker, regulatory body BGMEA, BKMEA, policy-making body, government, and factory authorities can take into account the results of this study and determine future course of action to increase workers’ job satisfaction.

## 1. Introduction

There is a widespread understanding that an organization’s success, productivity, and workforce efficiency depend on having a satisfied workforce [1], and that job satisfaction is influenced by a number of factors [2]. Researchers have identified a variety of job satisfaction factors and their importance on workers’ job satisfaction for this reason. According to earlier studies, when an employee is happy, he will work as hard as possible to meet the organization’s goals [3,4,5]. Highly satisfied workers are typically more reliable and punctual, quite productive, more devoted, and happier in their personal lives [6]. Employees should be given opportunities for advancement for this reason, such as pay scales, employee participation in policy-making, and making attempts to boost organizational commitment [7]. The nature of the job, the way in which it is supervised, job security [8], recognition, and development are crucial factors for employees’ organizational commitment. Similarly, safety and good relationships with the supervisor and coworkers are the biggest sources of satisfaction [9,10]. It is also found that job satisfaction is associated with leadership and employees’ wellbeing. In the context of the nursing profession, ethical leadership affected the subjective well-being of nurses through job satisfaction both directly and indirectly [11]. Additionally, participation in pension, profit-sharing, and job security programs is positively correlated with job satisfaction [12,13], despite the fact that many studies have identified the opportunity for professional growth as the primary factor influencing job satisfaction [14,15].

The perceived association between what one expects and receives from one’s job and the extent of importance or values one attributes to it determines one’s level of job satisfaction or dissatisfaction [16]. However, these expectations and values vary across cultures and occupational levels, and garment industry workers are not exempt from this. Bangladesh’s apparel industry is crucial to the country’s economic and financial progression [17]. Employees in the garment industry are constantly under pressure to meet the demands of the global marketplace [18] because Bangladesh’s garment industry is one of the most demanding in the world [19]. The only thing that will encourage them to perform at a high level is job satisfaction [20]. Bangladesh is a developing nation that has many issues. One of the main issues is low industrial sector productivity, and the garment industry is no exception to that rule [21]. The garment industry is essential to the nation’s economic growth [22,23]. The happiness or unhappiness of garment workers has a significant effect on the quality and productivity of the industry [24]. There are many issues that clothing factories are currently dealing with. It must have valid justifications for it. Undoubtedly, one of the main reasons is economic, but human factors, such as satisfaction with a job and dissatisfaction, are equally significant, if not more [25]. In addition to becoming Bangladesh’s largest source of export revenue, the garment industry has also created a wealth of employment opportunities. Approximately 78% of the nation’s total export revenue comes from this sector [26]. Currently, Bangladesh is home to about 5000 factories producing ready-to-wear clothing. Over 4 million people were directly employed by them, and many more were indirectly employed [27]. The industrial sector, where more than two-thirds (3.20 million) of the workers are women, offers the most employment chances for women [28]. Reasons for the sector’s growth include low labor costs and skilled workers earning low wages [29]. The garment factories in our nation are plagued by a variety of labor-related issues. The degree to which employees are satisfied with their jobs is a good indicator of any adjustment issues [30]. Increased absenteeism, turnover, and other unfavorable behaviors may be caused by job dissatisfaction [31].

Keeping employees pleased benefits a business in many ways, including decreased turnover, greater productivity, increased profitability, a better brand impression, a devoted customer base, and a long-lasting effect on the macroeconomic indicators of the nation [32]. It is a great shock that the garment industry contributes the most to the economy, followed by remittances, but neither government nor non-government institutions are sufficiently aware of the fundamental rights of the garment workers who have allowed us to compete on the world market [19]. Therefore, there are some egotistical owners of the clothing industry who tend to pay low wages and make their employees work hard to increase their profit margins. Their workplace does not support their efforts [33].

The only way to solve many issues is with the aid of job satisfaction. However, there has not been much research completed on job satisfaction, job dissatisfaction, and the effects on Bangladeshi garment workers. Therefore, it is necessary to conduct in-depth research on garment workers in order to understand their attitudes toward and behaviors at work, which will help to improve measure satisfaction levels and lead to better labor relations in Bangladeshi garment factories. Although numerous studies on job satisfaction and its various aspects have been carried out in developed nations [5,19,20,31], Bangladesh and the garment industry in particular have seen a dearth of research. Due to the differences in socioeconomic conditions, studies completed in developed nations cannot be generalized to Bangladesh. Therefore, it is necessary to demeanor a study on the job satisfaction of garment workers. With this goal in mind, the current study has been created to conduct a research project on job satisfaction in the clothing industry. Therefore, it is essential to conduct a study on garment workers’ job satisfaction. A research project on job satisfaction in the garments industry has been developed with this objective in mind.

Could the degree of job satisfaction vary depending on certain factors? Our research’s main question is this. This empirical analysis, which is structured on three levels, aims to provide an answer to that query. On the first level, we looked at the key aspects of both jobs among Bangladeshi garment workers, emphasizing how personal and professional factors affect employees’ job satisfaction levels, organizational levels, and the depth of their interpersonal and professional connections. This essay makes several contributions to the literature on sustainable HRM. First, it examines how two key aspects of sustainable HRM—employee personal and professional factors related to the job—affect two major findings of sustainable HRM (job satisfaction), narrowing the gap in employment opportunities. The paper also makes a first attempt to look into how new types of influences are affecting job satisfaction.

Despite having a significant impact on reducing poverty, increasing employment rates, and generating foreign exchange in Bangladesh, the garment industry is beset by employee discontent. A number of studies have been conducted on the human resource management, social, economic, and political aspects of the garment industry [15,18,34,35]; however, only a small number of them have focused on the personal and professional aspects of job satisfaction. Therefore, we chose the title “Assessment of both Personal and Professional Aspects to Measure Job Satisfaction Levels among Garment Workers; Empirical Evidence from Bangladesh” for the current study.

## 2. Literature Review

### 2.1. Job Satisfaction

One of the more complicated topics, job satisfaction includes a wide range of emotions and circumstances. One of the workplace behaviors is job satisfaction, which is a sense of fulfillment derived from job experience [36]. Job satisfaction was described by Nduati and Wanyoike [37] as having positive and emotional views regarding one’s work. Job satisfaction, according to Abdulkhaliq and Mohammadali [38], is one of the elements of a professional viewpoint and might be useful in keeping managing employees. The amount of happiness a person experiences as a result of their work is referred to as their level of job satisfaction [39]. As a result, one of the subjects that organizational psychology researchers have studied the most extensively is job satisfaction [40]. Job satisfaction, in the words of Locke [41] is the favorable and pleasurable emotion that arises from an assessment of one’s position or work history. Work happiness is determined by a job’s appeal [34]. A person feels satisfied with their job when they meet expectations at work. Job satisfaction is also an outcome of a joyful mental state that yields excellent job roles [42]. Helmi and Abunar [43] revealed that someone is happy with their work when they work in a situation like this and meet expectations. Breaugh et al. [44] stated that a person’s desires for their work and the benefits they actually receive from it determine job satisfaction. Moreover, the contented state of mind that satisfies work requirements adds to job satisfaction [45,46]. People who are content and joyful in their jobs do better than those who are not [47]. Emotional factors that affect job satisfaction include pleasure, happiness, passion, enthusiasm, and love [3,48]. According to the definition of Reig-Botella et al. [49] job satisfaction is viewed as a confluence of psychological and environmental factors that can lead someone to genuinely express satisfaction with the work completed. In order to support this definition, consider how much job satisfaction is represented by what makes people feel satisfied [50]. Stritesky [51] described that there are two types of job satisfaction: intrinsic and extrinsic. Extrinsic job satisfaction refers to characteristics of a job that are unrelated to the actual job, such as pay and organizational management, whereas intrinsic job satisfaction refers to responses to characteristics of the actual job, such as expertise and autonomy. A positive outlook is on a job that comes from analyzing and assessing its components. Positive attitudes toward their jobs are associated with higher levels of job satisfaction, whereas negative attitudes are associated with lower levels of job satisfaction.

### 2.2. Job Satisfaction and Personal Aspects

Job satisfaction varies from person to person. Although the workforce working in the same organization enjoys similar facilities, titles, salaries, and allowances, they may not all be satisfied and dissatisfied at work. Some are satisfied and some are dissatisfied, this is the diversity of the human mind. One of the determining factors of job satisfaction in the workplace is personal aspects [52]. Employee age, experience, marital status, gender, and employee level affect job satisfaction. This has been proven by several studies [53,54,55]. Lange [56] and Minh-Quang Duong [57] found a meaningfully constructive influence of demographic factors on job satisfaction. According to Reig-Botella al. [49], job satisfaction is also higher for older workers and those with a lot of experience. The level of job satisfaction is relatively low for employees with higher education. In his research, Reuben [52] found that there are no gender differences that significantly affect job satisfaction. He also found that respondents’ age and experience have a positive impact on their enjoyment at work. Age and experience have a significant impact on workplace satisfaction, according to the study by Islam and Akter [18], who found no evidence of a positive effect from other personal factors, i.e., gender, relationship status, and job title [58]. According to Nazneen et al. [59], Bangladeshi garment workers’ job satisfaction is significantly influenced by socio-demographic factors such as age, education, status, and income.

The less engrossed they are in their work and the less concerned they are about the requirements of their job satisfaction; the highly educated workers are less satisfied [50]. Previous studies have demonstrated a strong relationship between employee satisfaction and age, with older workers reporting lower job satisfaction and less concern for the various aspects of their work [60]. Since they are more satisfied than young workers [51] many institutions are intent on keeping them on because of the collective knowledge and expertise of these workers [61]. This finding can be connected to research in the context of China’s urban workforce. The research found that new-generation migrants in China are less satisfied with their jobs and lives than first-generation migrants, despite having higher incomes. The research found that workforce aspirations rising faster than income in China’s fast-growing urban economy [62]. The achievement of personal goals, mental and physical health, long-term relationships, professional development, and personal security are just a few of the factors that contribute to successful aging at work. According to Simarmata et al. [63] and Omar et al. [64], other factors such as matrimonial status, gender, experience, and education have not been found to have any statistically significant effects on job satisfaction levels. Employee position and age also have an impact on job satisfaction levels. The only significant correlation between demographic factors and job satisfaction, according to Baeza et al. [65], is gender. Employees’ levels of job satisfaction vary depending on their age cohort [66]. According to Sánchez and Puente [67], personality, culture, and demographic traits are all personal traits that have no bearing on job satisfaction. According to Abdulkhaliq and Mohammadali [38], having children, a marriage, and academic credentials can all affect work satisfaction. Nguyen et al. [68] found that there is no discernible relationship between satisfaction and any of the control factors, such as age, gender, or educational attainment. Mesurado et al. [54] revealed that demographics’ effect on job satisfaction is considerably good such as gender, marital status, and designation, had no discernible beneficial effect. Numerous empirical studies have demonstrated the impact of elements connected to work on job satisfaction [35,69,70]. According to Kampf and Hernández [71] a number of studies have looked at demographic parameters to predict labor satisfaction; however, Gazi [72] discovered that, with the exception of gender, there is no substantial association between demographic characteristics and job satisfaction. Reig-Botella et al. [49] demonstrated a significant relationship between job satisfaction and age, education level, and environmental risk. Additionally, the relationship between environmental risk and job satisfaction, and organizational commitment are positively correlated. Additionally, older workers and those with less education are more committed to their jobs. The level of job satisfaction is lower for employees with higher education. Therefore, the present study postulates the following hypothesis:

**H1.**  *There is no significant influence of personal aspects (age, education, experience, income, gender, and marital status) on the overall job satisfaction of the respondents.*

### 2.3. Job Satisfaction and Professional Aspects

Among the influencing factors of job satisfaction, the role of the professional aspect is outstanding. Satisfaction also depends on the achievement of professional aspects, which means that these professional aspects are the main sources of job satisfaction. Employees are satisfied-dissatisfied considering the benefits they receive from their jobs. For example, if wages are high, satisfaction is high, but if there is no job security, dissatisfaction is at its peak. The present study attempts to determine the level of satisfaction by focusing on 11 professional aspects (i.e., pay, promotion, job status, job security, working condition, relation with colleagues, participation in decision making, autonomy in work, open communication, behavior of boss and recognition) that have been and are being extensively researched. The following literature reviews were reviewed to determine the relationship between job satisfaction and professional aspects.

Shan et al. [73] revealed that employee job satisfaction is significantly and adversely impacted by some professional factors, including working conditions, relationships with coworkers, and job status. More than 50% of garment workers in Bangladesh are satisfied with their jobs according to Asikullah’s [22] study on the subject. Furthermore, significant statistical predictors of employees’ job satisfaction include job security, working conditions and decision-making procedures, the lack of paid time off and some other financial incentives, the failure to consider personal interests, ineffective canteen facilities, and rewards and recognition. According to Chandrasekara [74], professional factors have a positive and significant correlation with employees’ job satisfaction. With the exception of promotional opportunities, all the factors showed a marginally positive and significant relationship. Erro-Garcés and Ferreira [75] found that similar to contract terms like pay increases and contract duration, workplace environmental factors are shown to play a significant role in explaining job satisfaction. Although monthly income is one of the best indicators of job satisfaction [76], keeping employees happy requires more than just high pay. Job security, working conditions, having high compared earnings, functional flexibility, and high involvement management practices are job and organizational characteristics that are positively related to job satisfaction [77]. Syed and Mahmud [19] found that the three additional factors—salary amount, prompt payment of salaries, and employee advancement policies—also have a big impact on workers’ satisfaction. The relationship between job satisfaction and the workplace, employees are more likely to enjoy their work when they feel comfortable. According to Hu et al. [78] and Haleem et al. [79], factors such as management chic, salary, working conditions, opportunities for training, performance reviews, and participation in decision-making have a big impact on how satisfied workers are with their jobs. Arslan [80] combines three types of exploitation—financial, physical, and psychological—that lead to labor unhappiness in Pakistan’s garment industry. According to Hechanova and Manaois [81], employee attitudes in the Philippines as well as organizational norms and controls are all influenced by ethical leadership that is subject to job satisfaction. Employee satisfaction with supervision is positively correlated in Malaysia with reward, referent, and expert powers [82]. Additionally, coercion and power have a negative impact on how satisfied people are with supervision [83]. In China, there is a significant and positive correlation between high-performance work systems, working environments, and job satisfaction [84]. Asma et al. [85] investigate the level of job satisfaction among Bangladeshi garment industry workers. The study demonstrates how professional factors like working conditions, pay, job security, and stress have a big impact on how happy employees are at work in this industry.

Yuan et al. [86] found that a much lower percentage of workers were happy with their current position. According to the study, factors that were more significant for workers’ overall job satisfaction than working conditions, job status, autonomy at work, involvement in management, and open communication included hours of work, extra pay, the reward for good work, organizational policies, promotional opportunities, and good relationships with coworkers [87]. Personal characteristics such as age, experience, marital status, income, education, and skill did not significantly affect how satisfied employees were with their jobs overall in the Savar area of the Dhaka districts [28]. According to Chan [88], there is a positive correlation between certain aspects of job satisfaction, such as pay, supervision, a fair promotion policy, operating procedures, access to other financial resources, opportunities for open communication, and relationships with coworkers. Job satisfaction and productivity predictors were found to be positively correlated by Shi, Gordon, and Tang [89]. According to Kosec et al. [90], job satisfaction is significantly impacted by the workplace environment. Better facilities are essential for organizations to operate efficiently, and a worker’s prime concern is their workplace. Bossler and Broszeit [91] found a significant absolute rise in the affected employees’ pay satisfaction. Only a small impact on all other job satisfaction factors can be inferred from the modest increase in job satisfaction, which is primarily driven by changes in pay satisfaction. The behavior of the boss and supervisor has a greater impact on organizational commitment and job satisfaction than task-oriented leadership behavior does on turnover intentions [92,93,94]. Okikiola [32] studied the effect of work environment and employees’ satisfaction. His investigation’s findings demonstrated a strong connection between the workplace environment and job satisfaction among employees. They also demonstrate a connection between job satisfaction and welfare benefits. The research also shows a strong correlation between employee job satisfaction and job security. Raziq and Maulabakhsh [95] discovered that there is a correlation between a favorable work environment and a worker’s job satisfaction. Alauddin et al. [96] conducted a study to determine the elements influencing Bangladeshi garment industry workers’ job satisfaction. They discovered that job security, salary, promotions, housing options, and supervisor behavior have the biggest effects on job satisfaction in the apparel industry. However, transportation infrastructure and opportunities for training and development have less of an impact on employee job satisfaction. Rahman et al. [45] discovered that of the eight factors, three—attractive salary, good job security, and better working conditions—have a strong impact on employees’ job satisfaction in Bangladesh’s ready-made garments industry, while the remaining factors—recognition and reward, supervisor behavior, decision-making opportunity, communication system, and training facilities—have a moderate impact. According to Bashir et al. [97], there is a strong correlation between an employee’s pay, relationship behavior, compensation issues, and training and career growth factors and their job satisfaction. The most significant variables that positively impacted worker job satisfaction were pay and compensation. Babalola and Ishola [98] and Serreqi [99] have chosen three factors that influence job satisfaction, such as benefits and pay supervision, and development and training. Employee engagement and working conditions have a significant positive impact on job satisfaction, according to Díaz-Carrión et al. [100]. Additionally, they demonstrated that workers are loyal to their companies and continually look for new, more effective ways to add value. Numerous empirical studies clearly notified the effect of the behavior of bosses on job satisfaction [69,70,101,102]. De Carlo [101] studied on impact of the behavior of bosses on job satisfaction. The results of the study demonstrated significant effects of boss behavior on job satisfaction. Sutherland [103] studied on effects of job status on job satisfaction. He found that the relationship between a person’s job status and job satisfaction is typically positive and occasionally statistically significant for self-employed people.

Khan and Mishra [104] revealed that job promotion affects employee job satisfaction in Oman. Employees of Oman at Makasar Government Region are currently considered to have a high enough satisfaction with promotional opportunities. Rahaman and Uddin [105] indicated that job training (JT) and promotion (PRO) both have a favorable effect on the level of job satisfaction among SME employees. Otto et al. [106] looked at the effects of pay increases and promotions to managers on job satisfaction. They discovered that promotions had a short-term, positive impact on job satisfaction but lost that impact after a year. Furthermore, men were more affected by promotions than women in terms of job satisfaction. According to a different study, the main factor influencing job satisfaction is job security [107,108]. In Bangladesh, a study on the satisfaction of garment workers was conducted by Islam et al. [25]. They discovered that there is a significant relationship between workers’ job satisfaction and their pay and benefits, supervisor behavior patterns, work and family life, and other factors. According to Rana [109], human resource management practices like teamwork environments, job autonomy, and leadership behavior all have a positive and significant relationship with job satisfaction.

The type of work, chances for advancement, accessibility towards further education, teamwork, coworkers, line managers, salary, and position within the organization are among the variables that affect job satisfaction, according to Bakotić [110]. Working conditions and job satisfaction were found to be significantly positively correlated by Joshi [24]. Several studies showed the effects of participation in decision-making on job satisfaction. All studies concluded that participation in decision-making positively and significantly correlated with job satisfaction [111,112,113]. Thus, we proposed the following null hypothesis:

**H2.**  *The respondents’ overall job satisfaction is not significantly impacted by the particular professional aspects (pay, promotion, job status, job security, working condition, the behavior of boss, open communication, autonomy in work, recognition, participation, and relation with colleagues).*

**H3.**  *There is no discernible difference in job satisfaction between employees and supervisors.*

## 3. Methods and Procedures

### 3.1. Survey Administration and Sampling

The majority of garment factories in Bangladesh are located within the city limits of Dhaka, Chittagong, Narayangangj, and Gajipur. In Bangladesh, three types of garment factories are currently in operation: woven, knit, and sweater. To select the sample factories for the current study, the BKMEA (Bangladesh Knit Manufacturers and Exporters Association) and BGMEA (Bangladesh Garment Manufacturers and Exporters Association) provided two lists of all garment factories. From these lists, twenty-five garment factories were chosen at random as a sample, with ten (10) woven, twelve (12) knit, and three (3) sweater factories from the districts of Gazipur, Narayanganj, and Dhaka.

Generally, there are three sections in garment factories such as cutting, sewing, and finishing. To obtain a representative sample, 350 employees were chosen from above 25 factories, which would include 114 supervisors and 236 workers, and 3 sections of each were considered.

Table 1 demonstrates that 67.4% of respondents were workers and the remaining 32.6% were supervisors. On the other hand, 45.7 percent of the respondents were working in woven garment factory and the rest 45.7 percent and 8.6 percent respondents were working in knit and sweater garment factories, respectively.

Ten woven garments were chosen on a random basis for the purpose of the present study. In woven garment factory, the number of employees varied from 200 to 2000. The capital investment in such industries varied from BDT 3 million to 40 million. Woven garment industries’ production capacity varied from 15,000 to 200,000 pieces per month. They, generally export their products to U.S.A., Canada, and European Countries. Woven Garment contributes earn 74.25% of the total garment export earnings of the country [27].

Twelve knit and three sweater garments were selected on a random basis for the purpose of the present study. In knit and sweater garment factories number of employees varied from 150 to 1000. Their capital investment varied from BDT 2 million to 20 million (without composite knitting unit). The production capacity of the knit and sweater garment varied from 100,000 to 500,000 pieces per month. Knit and sweater garments contribute 25.76% (knit 18.99% and sweater 6.76%) of the total garment export earnings of the country [27].

### 3.2. Measuring Instruments Used

The information collected from the subjects of the current study fall under the job satisfaction and its degree among the level of employees, impact of importance of job facets, and satisfaction/dissatisfaction with specific personal and professional aspects of job.

#### 3.2.1. Brayfield–Rothe Job Satisfaction Measurement Scale (JSMS)

To measure the overall job satisfaction of the subject, the Bengali version of the Baryfied–Rothe Scale [114] was used. This is a questionnaire, consisting of 18 items dealing with an individual’s feeling towards his job as a whole. Each item could be replied by checking any one of the five possible answers: “strongly agree”, “agree”, “undecided”, “disagree” and “strongly disagree”. The items were selected in such a way that the satisfied end of the scale was indicated by “strongly agree” and “agree” for the one-half of the items and by “strongly disagree” and “disagree” for the other half. The neutral response was undecided. The items at the satisfied end receive 5 points for “strongly agree”, 4 for “agree”, 3 for “undecided”, 2 for “disagree” and 1 for “strongly disagree”. On the contrary, the items for the dissatisfied end receive 5 points for “strongly disagree”, 4 for “disagree”, 3 for “undecided”, 2 for “agree” and 1 for “strongly agree”. The possible range of scores is therefore 18 to 90 points. The neutral point is at 54. A total score on or above the neutral point represents job satisfaction and a score falling below this point represents job dissatisfaction.

Brayfied and Rothe [114] reported a split-half reliability coefficient of 0.87 for this scale for a sample of 231 female clerical workers. Concerning validity, they recounted a correlation of 0.93 between the Brayfied–Rothe Scale and the Hoppock Blank. Inayat et al. [1] reported correlation coefficients reaching from 0.48 to 0.75 between the Baryfied–Rothe Scale and the Job Descriptive Index (JDI) in three dispersed samples. Khaleque [115] reported a correlation coefficient of 0.63 between the Baryfied–Rothe Scale and the Job Descriptive Scale (JDI) [116] on three separate samples (Table A1).

#### 3.2.2. Questionnaire for Measuring Satisfaction with Professional Aspects (QMSPA)

A questionnaire was erected to measure satisfaction with 11 specific professional aspects of the job. These items were comprised in the questionnaire for covering different but representative areas, which have been suggested by several investigations to affect employee attitudes [34,37,115,117]. The respondents would specify their satisfaction or dissatisfaction with each of these specific aspects by checking either “Yes” or “No” responses (Table A2).

### 3.3. Pilot Survey

The original version of the job satisfaction scale [114] for measuring job satisfaction was in English. Since the mother language of the employees of the current study was Bangla and average schooling of the respondents was 6.66 years, it seemed necessary to administer the questionnaire in Bangla. Khaleque [115] translated the Brafield–Rothe Scale into Bangla and used that version after proper reliability test. For the purpose of the present study, that translated version of the scale was used. The questionnaire, with the above objectives in view, was administered on a sample of 25 employees of 3 garment factories. The questionnaire and its responses were deemed to be extremely satisfactory in terms of the subjects’ understanding of the questions.

### 3.4. Data Analysis

The Statistical Package for the Social Sciences (SPSS) version 23 was used to analyze the data (IBM software Version 23.0, Chicago, IL, USA). Descriptive statistics measures like frequency, mean, and standard deviation, z-test, were used to describe participant characteristics, job satisfaction. To identify the factors influencing job satisfaction level, Pearson’s correlation regression analysis was used, with adjustments for employees’ personal aspects and professional aspects. A significance level of <0.05 was used for all statistical analyses.

### 3.5. Procedures of the Study

**Figure 1:**
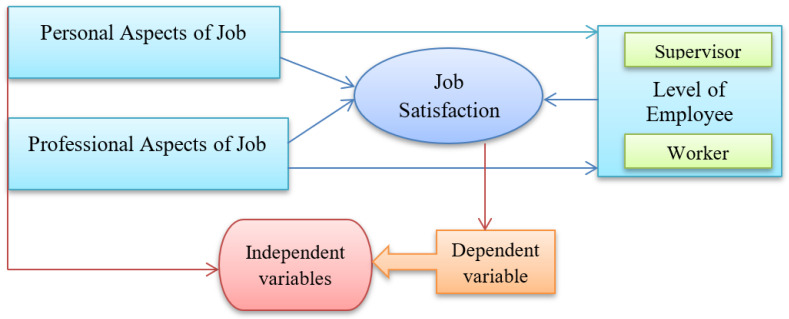
Research framework.

For the purpose of the final study, twenty-five (ten woven, twelve knits, and three sweaters) were selected on a random basis. Three districts were purposively selected to collect the data (viz. Dhaka, Narayanganj, and Gazipur districts). Each of the chosen garment factories received the required written consent from the relevant authority. After obtaining permission, each respondent was personally contracted, and data were collected from each one of them after convincing them of the study’s goals. The relevant factory authority provided a suitable room for the sittings to be held in. The interviews were conducted throughout working hours, at lunchtime, and even after hours. Data were gathered by the researchers themselves between June 2021 and July 2022. The respondents received the necessary clarifications whenever they encountered a problem. 350 subjects in total were chosen and interviewed. Less than two-year veterans were not allowed to work there. The interviews were conducted throughout working hours, at lunchtime, and even after hours. The time it took for each subject to complete the information needed to fill out the questionnaire was approximately 30 to 40 min. All the answer sheets were checked one by one and incomplete answer sheets were excluded from the study. The researcher himself tabulated the data. Before tabulation, a code plan was prepared, and data were transferred into a code sheet. Based on the previous literature review the authors developed a research framework (Figure 1).

## 4. Data Analysis and Results

### 4.1. Demographic Profile

According to Table 2’s findings, 49.1% of the supervisors were between the ages of 22 and 29. On the contrary hand, the workers who made up a large proportion (44.5%) were between the ages of 14 and 21. Additionally, it was noted that the supervisors’ average age (27.15) was higher than the workers’ (23.14). The results also show that the supervisors’ mean age was significantly older than the workers’. So, it is obvious that the Bangladeshi garment industry was dominated by the youngest supervisors and workers (aged 14 to 21).

Table 2 shows that 12.3% of the employees were completely illiterate, and the highest number of supervisors (28.1%) was from 12 years (H.S.C.) of schooling group and the highest percentage among the workers (45.3%) were from (class I–V) one to five years of schooling. The table also demonstrates that the supervisors’ average educational level was higher (9.44) compared to the workers (5.31). The findings also show that the supervisors had significantly more education than the employees did. Additionally, it appeared from Table 2 that the supervisors’ mean income (8845.45) was higher than the workers’ (7514.08). Additionally, it was noted that the supervisors’ and employees’ primary income range fell between TK. 5000 and 8000. Table 2 shows that the group with the greatest representation of supervisors (48.2%) and employees (41.5%) was that with 2–5 years of experience. Table 2 suggests that the mean experience of the workers was slightly higher (6.16 years) than that of the supervisors (6.85 years), which is not statistically significant. Further Table 1 shows that 67.4% of participants were workers and the remaining 32.6 percent were supervisors. On the other hand, 45.7 percent of the respondents were working in woven garment factories and the rest 45.7 percent and 8.6 percent of respondents were working in knit and sweater garment factories, respectively. Again, Table 2 shows that 77.4 % of the respondents were paid on a time rate basis and the rest 22.6% were paid on a piece rate basis. On the other hand, among the workers, 66.5% were paid on a time rate basis and the rest 33.5% were paid on a piece rate basis. It may be mentioned here that, the basis of payment of all the supervisors was time basis which means payment of piece rate basis was absent for supervisors in garment factories. Table 2 shows that 44.00% of the respondents were women and 56.00% of the respondents were men. There were 73.68% male supervisors and 26.32 percent female supervisors. Again, of the workers, 52.54 were female and 47.46% were men.

### 4.2. Statistical Analysis and Results

The present study deals with the results and analyses of data comparing the garment employees (woven, knit, and sweater) and with the supervisors and workers and the basis of satisfaction. Results and analyses of data on the variables are presented in the following sections:

Since age, experience, education, and income have a significant influence on the overall job satisfaction of the employee, so the respondents were classified into two groups—high and low groups to see the stimulus of these aspects on the overall job satisfaction of the respondents. The respondents below and above the median age, experience, education, and income were categorized as lower and higher groups, respectively. The subjects were also categorized as male and female and married and unmarried. The mean differences in job satisfaction according to the above personal factors of the respondents are shown in Table 3.

Table 3 shows that only one out of seven z-ratios was significant, and the other six were not significant. The table indicates that education, income, experience, type of organization, sex, and marital status had no significant influence on overall job satisfaction. On the other hand, age had a significant impact on job satisfaction. The results further indicate that job satisfaction was greater among the high-age group than that of the low-age group.

To examine the influence of specific professional aspects on overall job satisfaction, respondents were categorized into satisfied and dissatisfied groups on each aspect of the job. Z-test was applied to compare the overall mean job satisfaction score of the two groups and the results are shown in Table 4.

Table 4 shows that the mean overall job satisfaction of satisfied people who responded was significantly greater than those who were dissatisfied with particular aspects of their job, with the exception of two job factors, namely promotional opportunity and relationship with colleagues. The findings indicate that overall job satisfaction differed significantly with satisfaction and dissatisfaction of the specific job factors.

Table 5 compares the significant differentiation, respectively, satisfaction and dissatisfaction with particular profession aspects.

According to Table 5, an expressively higher portion of respondents were dissatisfied with about their job security, pay, decision-making participation, promotional opportunity, autonomy at work, and job status. A significantly higher proportion of respondents, however, were satisfied with their boss’s behavior, working conditions, open communication, and recognition for work.

The z-test was used to determine whether there is a significant difference in the mean job satisfaction of supervisors and workers, and the results are shown in Table 6.

There was a significant average difference in job satisfaction between supervisors and workers, according to the results of Table 6. According to the findings, supervisors were significantly more satisfied with their jobs than workers.

Table 7 compares whether there is a difference in satisfaction and dissatisfaction with specific job factors based on employee level (supervisors and workers).

The upshots in the Table 7 reveal that the response patterns of supervisors and employees differed significantly regarding their promotional opportunity, job status, the behavior of bosses, open communication, autonomy in work, recognition system, and participation in management. The rest of the four aspects (i.e., pay, job security, working condition, and relation with colleagues) did not differ significantly. The findings of the table suggest that most of the supervisors felt job insecurity, while workers’ felt that there was no scope for participation in management.

Table 8 displays the results of a comparison of respondents’ satisfaction and dissatisfaction with specific job factors based on the type of organization.

A comparison was made to examine the satisfaction and dissatisfaction with specific professional aspects of the job between the employees of woven garment factories and knit and sweater garment factories. The results reveal that there were significant differences in their responses on promotional opportunity, job status, job security, the behavior of bosses, open communication, and recognition system. There was no difference in the response pattern on the rest of the aspects between respondents of these two types of organizations. The results show that significantly higher numbers of the respondents in woven garment employees were dissatisfied with their promotional opportunity as compared to knit and sweater garment employees. On the other hand, higher numbers of the respondents in knit and sweater garments factories were dissatisfied with their job security as compared to woven garment employees. While a significantly higher number of respondents in knit and sweater garment employees were satisfied with the behavior of their boss, and recognition for good work open communication (Table 8).

The individual contributions of several dependent variables to one dependent variable: job satisfaction was investigated, as well as the interrelationships between the variables. Table 9 and Table 10 show the outcomes of inter-correlation and individual contribution.

Table 9 depicts the interrelationships between the variables of age, education, total income, work experience, and job satisfaction for all respondents (N 350) and are investigated to determine the feature and magnitude of the correlation that exists between those variables.

The results of the correlation matrix (Table 9) show that all but one variable selected was significantly correlated with job satisfaction and was relevant for explaining variation in the level of satisfaction. The table also suggests that age had a significant positive correlation with the job satisfaction of the respondents. The table further indicates that job satisfaction had no significant correlation with the rest of the variables (i.e., education, total income, and work experience).

Table 10 shows that after adhering to the given statistical criteria, only one of the independent variables was entered into the equation, i.e., age, and its multiple R and R square values were 0.113 and 0.013, respectively. This indicates that age was the only predictor of job satisfaction having a contribution of about 1.3 percent. The equation suggests that age had a significant contribution to job satisfaction (beta 0.113).

The results in Table 11 show that there was a negative significant correlation found among job satisfaction and job status, job satisfaction and behavior of boss, and job satisfaction and open communication. On the other hand, a positive significant correlation had been found among job satisfaction and pay, job satisfaction and promotion, job satisfaction and working conditions, job satisfaction and job security, and job satisfaction and participation in decision-making.

Table 12 demonstrates that only five of the independent variables (professional aspects), including pay, promotion, job security, working conditions, and participation in decision-making, were included in the analysis after adhering to the provided statistical criteria. The multiple R and R square increased with the addition of the independent variables (professional aspects). This shows that the best groups of predictors of job satisfaction, with a combined contribution of about 68.2%, were pay, job security, working conditions, promotion, and participation in decision-making. Their multiple R values were 0.518, 0.609, 0.688, 0.724, and 0.822, respectively. It was discovered that professional factors, such as independent pay, promotion, job security, working conditions, and participation in decision-making, all had an impact on job satisfaction (beta 0.188, 0.169, 0.205, 0.158, and 0.123). According to the equation, certain professional factors (independent variables) may have made a significant impact on job satisfaction.

## 5. Discussion

In this section, the findings of the current study are examined in relation to the previously set two hypotheses in the light of the beforehand stated goals of the current investigation and after a review of the literature. The results of the current study will be compared to each hypothesis.

The results suggest that out of six personal aspects, only one aspect (such as age) had a significant influence on overall job satisfaction and the rest five aspects (such as education, income, experience, sex, and marital status) had no significant influence on the overall job satisfaction. Therefore, the results partially confirmed the first null hypothesis. The direction of the results indicated that the respondents with higher age were significantly more satisfied than those of the lower age group (Table 3). Correlational results also indicated a significant positive correlation between age with overall job satisfaction (see Table 9). The findings revealed that employee level had a significant effect on job satisfaction, with supervisors having a significantly greater mean job satisfaction scoring rate than workers (Table 6). Several studies have also found that higher-level employees are more satisfied than lower-level employees. For instance, Lam et al., 2020 reviewed a vast amount of literature and found that top-level employees were more satisfied than those of bottom-level employees. Steel [118], Yainahu et al. [119], Yoo [120], Kim and Cho [35], and Qureshi et al. [121] likewise noticed similar consequences, which confirmed the findings of the current study.

Employees at lower levels were generally more dissatisfied than higher-level employees, according to Stritesky [51]. He contended that higher-level employees were more satisfied than lower-line employees because they had more prospects to satisfy their ego needs, more status, more pay, and self-direction than lower-level employees. Furthermore, they were given more power and duty than lower-level employees. The current study also found that supervisors were more satisfied than workers with their working conditions, boss behavior, open communication opportunities, autonomy at work, recognition for good work, and participation in management (see Table 7). The present study further revealed that these factors had a significant influence on the overall job satisfaction of the respondents (see Table 3 and Table 4) which might be the cause for higher job satisfaction among the supervisors than that of the workers.

Several studies [35,49,50,54,58,64,69] conducted both at home and abroad discovered a significant favorable influence of personal factors on overall job satisfaction, confirming the current study’s findings.

The present study further suggested that there was a significant impact of the level of organization on overall job satisfaction. The results showed that job satisfaction was higher among the employees of oven garments as compared to their counterparts. It was also observed from Table 3 that married employees were higher satisfied than unmarried. Employees were more satisfied with the behavior of their boss, the opportunity for open communication, and recognition for good work. The fallouts of the current study further indicated that these issues had a significant confident influence on the overall job satisfaction of the respondents. It was found from the results that all the specific professional aspects had a significant influence on overall job satisfaction, except one factor such as the relationship with colleagues (see Table 5). Hence, the findings rejected the second null hypothesis. When comparing overall job satisfaction between supervisors and workers based on professional aspects revealed a significant influence on overall job satisfaction. The research found exceptions in the case of pay, job security, and working condition (Table 7). Pearson’s Correlation Coefficient results also confirm that professional aspects had a great impact on the job satisfaction of employees in the garment industry in Bangladesh (Table 11 and Table 12). The results further suggested that overall job satisfaction was pointedly higher among the satisfied respondents than that of the dissatisfied respondents. Several investigators, i.e., Asikullah [22]; Shan et al. [73]; Syed and Mahmud [19]; Arslan [80]; Hechanova and Manaois [81]; Shi, Gordon and Tang [89] also found a significant impact of professional aspects on the overall job satisfaction of the respondents, which inveterate the outcomes of the present study.

The socio-economic conditions of Bangladesh are different from those of other garment-producing countries in the world, so it is natural, that the results of research conducted in the context of Bangladesh will not be consistent with the results of research conducted in the context of other countries. There is a big difference, especially with western countries. However, the socio-economic conditions of Asian countries are almost similar. Overall personal aspects and professional aspects are universal in all countries. Depending on the country, the presence of these factors affecting the job satisfaction of workers is observed to varying degrees in different countries.

## 6. Conclusions

For an organization to grow and succeed, employee job satisfaction is a fundamental issue for retaining a productive workforce. Organizations must comprehend the employee attitudes that better motivate employees in order to increase productivity and achieve organizational goals. Job satisfaction is the main weapon to make a group loyal and motivated them. The purpose of the study is to assess the personal and professional variables that influence garment employee job satisfaction in a developing country covered Bangladesh. The present study’s findings suggest that only age had a significant influence on overall job satisfaction and the other five, i.e., education, income, experience, sex, and marital status had no significant influence on the overall job satisfaction of the respondents. Specific professional aspects factors, i.e., pay, job security, the behavior of bosses, job status, autonomy in work, recognition for good work, participation in decision making, communication with the boss, and working environment had a significant influence on the overall job satisfaction of the respondents. Between the personal and professional aspects, the latter plays a decisive role in producing job satisfaction for the respondents. Both the men and women workers’ apparent garment jobs are extremely stressful, but female respondents perceived significantly higher job stress than that of their counterparts. Among the independent variables such as age, experience, education, income, and job satisfaction, the contribution of job satisfaction to performance (dependent variable) was the highest. According to this study, the supervisor’s mean job satisfaction notch was significantly greater than the workers’. Between the high and low age groups, there were appreciably different average levels of job satisfaction. Employees who are older report being happier at work than respondents who are younger. Therefore, distinct programs for the current low-aged workers should be executed by the garment industry authorities to ensure maximum satisfaction. The groups of respondents with more experience and less education were found to be happier. In order to pay distinctive attention to, keep, and encourage experienced and highly educated respondents, the garment authorities may do so. Job satisfaction has varied significantly across the apparel industry. Both worker levels and two-way interactions in the garment industry were not statistically significant. So, the results obtained from the analysis show that only age affects job satisfaction, but higher-aged workers are more satisfied than low-aged workers. Hence, garment owners should take measures to ensure maximum satisfaction for young workers. In addition, it can be seen that in the case of other personal factors, the impact on job satisfaction is not seen significantly, but experienced and highly educated workers are less satisfied. The concerned authorities should take incentive measures to retain highly educated and experienced workers in the organization and come forward to improve their living standards by increasing wages.

The degree of satisfaction among employees heavily influences productivity. Additionally, it is challenging to satisfy all employees by offering the same things. Some employees might be content with some professional factors, but they might not give much thought to how they are treated at work. The garments industry should therefore take into account each of these factors. All of the factors that contribute to employee satisfaction must be taken into account by the management. Professional aspects, i.e., pay promotion, job security, job status, recognition, kith and kin with colleagues, participation in decision-making, open communication, autonomy in work, and the behavior of bosses have a significant impact on workers’ job satisfaction in Bangladeshi readymade garments industry, while the other factors have a moderate impact. The happiness of Bangladeshi readymade garment workers depends greatly on their pay, promotion, job security, working conditions, and participation in decision-making. This study places a priority on improving working conditions right away. As a result, the company needs to restructure its plan for improving the factory’s work environment to include better adequate care, health and hygiene facilities, and safety equipment. The garment industry pays its employees a low wage that is not advised by the government and is currently insufficient for the workers. Therefore, in order for the employees to have a better quality of life, the owners, management, and regulatory authority of the company should adhere to the government-recommended salary structure and a little more. The workers will be happier as a result of an increase in pay. There is no minimum opportunity for workers’ participation in management in the garment industry sector of Bangladesh. The opinion of the workers has no value, rather the workers are harassed in various ways. There is a trend of getting fired at the drop of a hat. Incidents of mental and physical abuse also occur frequently. Employees suffer from job insecurity. Job loss due to various reasons is a common phenomenon in the garment industry of Bangladesh. The cases of regular promotion of workers are rare, so the workers do not get the thought and opportunity to build a long-term career. So, the garment authority should provide job security to the workers first and give merit-based promotions so that they can build long-term careers in the garment industry. Workers’ views should be given importance. One of the best aspects of modern management is the opportunity for workers to participate in management. If there is such an opportunity, the workers are more committed to the organization, and the satisfaction increases. Garment industry authorities and management should consider the factors and evaluate the workers contributions. If the aforementioned demands of the workers in the ready-made industry can be met, this industry will establish a high foreign currency earnings country, and improved socioeconomic conditions of garments workers are all expected. The current study recommends that the government, the BGMEA, and the BKMEA implement appropriate strategies and measures to guarantee all of the aforementioned factors for the satisfaction of all Bangladeshi garment workers.

Some of this research paper’s strengths set it apart from others. Such research with garment industrial workers in Bangladesh is, to put it mildly, extremely uncommon. The study, which will be recognized as a landmark, was carried out by researchers in the mentioned field. There is an abundance of research on job satisfaction worldwide. Numerous studies have been, are being, and will be conducted in the future. Our research is more unique and fundamental as a result of the paucity of literature in the field related to the garment industry. The methodology of this study is yet another plus. The widely accepted measurement scale has been used for data analysis. Additionally, direct interviews are used to gather the data so that it can be pragmatically analyzed.

This research has limitations just like other research does. Twenty-five garment factories were included in this research, from which the sample was pinched, but not all of them. Instead of studying the entire garment factories, the researcher concentrated on the garment factories located in Dhaka, Gazipur, and Narayangonj districts. As a result, the researcher might not be able to extrapolate the findings to all the garment factories in the country. Consequently, only 350 out of the thousands of workers employed by the garment industry in Bangladesh were included in the study, which is another drawback of the study. The majority of the ready-made garment workers were reluctant to disclose their internal policies and provide responses due to the confidentiality and demanding work schedules, making it impossible to conduct a large survey and obtain a large sample size. The population’s geographic scope is another drawback of the study. Researchers were given less time than was necessary to ample the research, which had an impact on the collection of pertinent data from various sources. Researchers were enforced to use lesser sample sizes that hampered data collection due to a lack of funding and time. All of the nation’s state-owned sugar mills could be included in the population. More thorough research is therefore needed in this established field in Bangladesh.

A larger sample size, more personal and professional aspects, more garment factories from various districts of the country, a new advanced questionnaire, upgraded methods, and other tools for measuring various aspects of job satisfaction which can be more in number should be used in future studies using a framework alike to that of the present study in order to obtain more accurate results. With the use of contemporary research techniques, it is expected that subsequent researchers will be capable to create a genuine portrait of workers’ job satisfaction by accounting for the limitations of the current study, taking into account the significant number of respondents, and taking into account garments factories generally.

## Figures and Tables

**Table 1:** Distribution of samples based on organization type and employee level (N = 350).

Unit of Garment Industry	Level of Employee	Number	Percentage	Total
Woven	Supervisor	51	14.6	160 (45.7%)
	Workers	109	31.1	
Knit	Supervisor	55	15.7	160 (45.7%)
	Workers	105	30.0	
Sweater	Supervisor	8	2.3	30 (8.6%)
	Workers	22	6.3	
Total	S/W	350	100%	350 (100%)

**Table 2:** Demographic Information of the respondents.

**Age (in year)**	**Age group**	**Supervisor**		**Workers**		**Experience (in year)**	**Experience**	**Supervisor**		**Workers**	
	14–21	22	19.3%	105	44.5%		2–5 years	55	48.2%	98	41.5%
	22–29	56	49.1%	101	42.8%		6–9 years	37	32.5%	88	37.3%
	30–38	29	25.4%	27	11.4%		10–13 years	17	14.9%	35	14.8%
	30 & above	7	6.1%	3	1.3%		14 & above	5	4.4%	15	6.4%
	Total	114	100%	236	100%		Total	114	100%	236	100%
	Average	27.15		23.14			Average	6.16		6.85	
**Education Level**	**Education**	**Supervisor**		**Workers**		**Income (Monthly in BDT)**	**Income**	**Supervisor**		**Workers**	
	Illiterate	1	0.9%	29	12.3%		BDT 5000–8000	73	64.0%	175	74.2%
	Class 1–5	18	15.8%	107	45.3%		BDT 8001–11,000	35	30.7%	40	16.9%
	Class 6–9	26	22.8%	70	29.66%		BDT 11,001–15,000	6	5.3%	9	3.8%
	S.S.C.	27	23.7%	26	11.0%		BDT 15,001 & above	0	0%	12	5.1%
	H.S.C.	32	28.1%	4	1.7%		Total	114	100%	236	100%
	Degree & above	10	8.8%	0	0%		Average	8845.45		7514.08	
	Total	114	100%	236	100%		NB: BDT 95 = 1 USD				
	Average	9.44		5.31			As per the exchange rate of August 2022				
**Organization**	**Org. Type**	**Supervisor**		**Workers**		**Sex**	**Gender**	**Supervisor**		**Workers**	
	Woven	51	14.6%	109	31.1%		Male	84	73.68%	112	47.46%
	Knit	55	15.7%	105	30.0%		Female	30	26.32%	124	52.54%
	Sweater	8	2.3%	22	6.3%		Total	114	100%	236	100%
	Total	114	32.6%	236	67.4%		56% of the respondents were male 44% were female				
**Marital Status**	**Status**	**Supervisor**		**Workers**		**Basis of Payment**	**Payment**	**Supervisor**		**Workers**	
	Married	63	55.26	104	44.07		Time basis	114	100%	157	66.5%
	Unmarried	51	74.44	132	55.93		Piece basis	0	0%	79	33.5%
	Total	114	100	236	100		Total	114	100%	236	100%

**Table 3:** The respondents’ (N = 350) mean differences in satisfaction according to some personal characteristics (age, education, income, experience, type of organization, sex, and marital status).

Groups	Number	Mean	S.D.	S. Err. Mean	z	df.	Sig. (2-Tailed)	Mean Diff.
Low age (14 years) High age (30 years and above)	188	58.55	7.00	0.51	−2.26	348	<0.05	−1.71
	162	60.26	7.11	0.56				
Lower education Higher education	190	59.32	6.24	0.45	−0.078	348	N. S.	−0.059
	160	59.38	8.00	0.63				
Low income High income	232	59.25	7.70	0.51	−0.359	348	N. S.	−0.29
	118	59.53	5.73	0.53				
Low experience High experience	218	59.43	7.35	0.50	0.284	348	N. S.	0.222
	132	59.21	6.66	0.58				
Woven Garment Knit & Sweater Garment	160	59.62	7.98	0.63	0.668	348	N. S.	0.508
	190	59.11	6.25	0.45				
Male Female	196	59.71	7.15	0.51	1.11	348	N. S.	0.844
	154	58.87	7.01	0.56				
Married Unmarried	167	59.76	6.41	0.50	1.05	348	N. S.	0.254
	183	58.96	7.66	0.57				

Note: N. S. = Not Significant.

**Table 4:** Mean difference in job satisfaction according to the degree of satisfaction and dissatisfaction with some of the specific professional aspects (N = 350).

Specific Job Factors	Mean Overall Job Satisfaction Scores of the Respondents Who Are Satisfied with the Factors			Mean Overall Job Satisfaction Scores of the Respondents Who Are Dissatisfied with the Factors			z	df.	Sig. (2-Tailed)
	No.	Mean	S.D.	No.	Mean	S.D.			
Pay	19	64.32	7.54	331	59.06	6.97	3.19	348	<0.01
Promotion	105	58.53	5.65	245	59.69	7.61	−1.40	348	N. S.
Job status	133	58.27	6.01	217	60.00	7.62	−2.23	348	<0.05
Job security	12	64.58	8.14	338	59.16	6.99	2.62	348	<0.01
Working condition	305	59.64	6.52	45	57.36	10.00	2.02	348	<0.05
Behavior of boss	311	59.92	6.32	39	54.72	10.56	4.44	348	<0.01
Open communication	290	59.83	6.71	60	57.00	8.37	2.84	348	<0.01
Autonomy in work	117	62.21	6.42	233	57.91	6.99	5.58	348	<0.01
Recognition for good work	284	60.06	6.45	66	56.24	8.76	4.03	348	<0.01
Participation in decision making	59	63.83	5.18	291	58.43	7.08	5.56	348	<0.01
Relations with colleagues	344	59.38	7.04	6	57.17	9.97	0.76	348	N. S.

Note: N. S. = Not Significant.

**Table 5:** Composite chi-square regarding the satisfaction and dissatisfaction of respondents with specific professional aspects (N = 350).

Specific Aspect of Job	Numbers of the Satisfied Subjects	Numbers of the Dissatisfied Subjects	Chi-Square	df.	*p*
Pay	19	331	63.51	40	<0.01
Promotional opportunity	105	245	101.24	40	<0.01
Job status	133	217	82.52	40	<0.01
Job security	12	338	95.89	40	<0.01
Working condition	305	45	70.93	40	<0.01
Behaviour of boss	311	39	100.35	40	<0.01
Open communication	290	60	65.39	40	<0.01
Autonomy in work	117	233	68.98	40	<0.01
Recognition system	284	66	69.01	40	<0.01
Participation in management	59	291	86.94	40	<0.01
Relation with colleagues	344	6	38.90	40	N. S.

Note: N. S. = Not Significant.

**Table 6:** Mean difference in job satisfaction according to level of employees of the respondents (N = 350).

Group	Number	Mean	S.D.	z	df.	Sig. (2-Tailed)
Supervisors	114	60.49	7.53	2.12	348	<0.05
Workers	236	58.79	6.82			

**Table 7:** Composite chi-square analysis of respondents’ satisfaction and dissatisfaction with specific professional criteria based on their level (N = 350).

Specific Professional Aspect of Job	Supervisor (N = 114)		Worker (N = 236)		Chi-Square	df.	*p*
	Yes	No	Yes	No			
Pay	4 (3.51%)	110 (96.49%)	15 (6.36%)	221 (93.64%)	1.21	1	N. S.
Promotional opportunity	11 (9.65%)	103 (90.35%)	94 (39.83%)	142 (60.17%)	33.34	1	<0.01
Job status	26 (22.81%)	88 (77.19%)	107 (45.34%)	129 (54.66%)	16.56	1	<0.01
Job security	2 (1.75%)	112 (98.25%)	10 (4.24%)	226 (95.76%)	1.43	1	N. S.
Working condition	102 (89.47%)	12 (10.53%)	203 (86.02%)	33 (13.98%)	.82	1	N. S.
Behavior of boss	108 (94.74%)	6 (5.26%)	203 (86.02%)	33 (13.98%)	5.90	1	<0.01
Open communication	107 (93.86%)	7 (5.14%)	183 (77.54%)	53 (22.46%)	14.41	1	<0.01
Autonomy in work	59 (51.75%)	55 (48.25%)	58 (24.58%)	178 (75.42%)	25.51	1	<0.01
Recognition system	104 (91.23%)	10 (8.77%)	180 (76.27%)	56 (23.73%)	11.24	1	<0.01
Participation in management	51 (44.74%)	63 (55.63%)	8 (3.39%)	228 (96.61%)	93.76	1	<0.01
Relation with colleagues	113 (99.12%)	1 (.88%)	231 (97.88%)	5 (2.12%)	0.70	1	N. S.

Note: N. S. = Not Significant.

**Table 8:** Composite chi-square for satisfaction and dissatisfaction with specific professional aspects based on the type of organization (woven and knit and sweater garment, N = 350).

Specific Aspect of Job	Woven Garment		Knit & Sweater Garment		Chi-Square	df.	*p*
	Yes	No	Yes	No			
Pay	9	151	10	180	0.022	1	N. S.
Promotional opportunity	17	143	88	102	52.686	1	<0.01
Job status	34	126	99	91	35.099	1	<0.01
Job security	9	151	3	187	4.294	1	<0.05
Working condition	140	20	165	25	0.034	1	N. S.
Behavior of boss	131	29	180	10	14.512	1	<0.01
Open communication	122	38	168	22	9.058	1	<0.01
Autonomy in work	52	108	65	125	0.114	1	N. S.
Recognition system	119	41	165	25	8.823	1	<0.01
Participation in management	27	133	32	158	0.000	1	N. S.
Relation with colleagues	157	3	187	3	0.045	1	N. S.

Note: N. S. = Not Significant.

**Table 9:** Matrix displaying bivariate correlations between some selected independent variables (respondent age, education, total income, and work experience) and one dependent variable: job fulfillment (N = 350).

Variables	1	2	3	4	5
1. Age	1.000				
2. Education	0.099	1.000			
3. Total income	0.322 **	0.156 **	1.000		
4. Work experience	0.540 **	–0.231 **	0.420 **	1.000	
5. Job satisfaction	0.113 *	0.033	0.001	–0.020	1.000

* Correlation is significant at the 0.05 level (2-tailed). ** Correlation is significant at the 0.01 level (2-tailed).

**Table 10:** Step-wise multiple regression based on personal aspects: dependent variable job satisfaction.

Variable in the Equation	R	R^2^	F	*p*	Beta
Age	0.113	0.013	4.497	0.035	0.113

**Table 11:** Correlation matrix among dependent (job satisfaction) and independent (professional aspects of job) variables (N = 300).

Variables	1	2	3	4	5	6	7	8	9	10	11	12
1. Job satisfaction	1											
2. Pay	0.651 **	1										
3. Promotion	324 **	0.305 **	1									
4. Job status	−0.418 **	0.168 **	0.506 **	1								
5. Job security	0.135 **	−0.18 *	0.287 **	0.285 **	1							
6. Working condition	0.506 **	0.257 *	0.178 **	0.357 **	0.432 **	1						
7. Behavior of boss	−0.308 **	0.222	0.022	0.285 *	−0.129 **	0.725 **	1					
8. Open communication	−258 **	0.225 *	0.001	0.076	0.008	0.187 **	0.528 **	1				
9. Autonomy in work	0.255	0.009	0.128	0.364	0.396 **	0.236 *	0.140 *	0.156 **	1			
10. Recognition for good work	309	0.093	−0.189 **	0.352 **	0.058	0.315 **	0.281 **	0.332 **	0.148 *	1		
11. Participation in decision making	369 **	−850	0.064	0.008	0.185	258 **	0.180 **	0.149 **	0.212 **	0.354 **	1	
12. Relation with colleagues	0.580	−0.085 **	−0.087	0.025	−0.074	0.055	0.031	0.012	0.044	0.014	0.211 *	1

Note * *p* > 0.05, ** *p* > 0.01.

**Table 12:** Summary of step-wise multiple regression: dependent variable job satisfaction.

Variables in the Equation	R	R^2^	F Value	*p* Value	Beta
Pay	0.518	0.337	78.25	0.000	0.188
Promotion	0.609	0.441	70.20	0.000	0.169
Job security	0.688	0.502	55.98	0.000	0.205
Working condition	0.724	0.594	48.72	0.000	0.158
Participation in decision making	0.822	0.682	31.54	0.000	0.123

## Data Availability

Not applicable.

## Appendix A

**Table A1:** Overview of Baryfied-Rothe’s job satisfaction scale.

No. of Items	Statements	Reference
01.	My job is like a hobby to me.	JSMS
02.	My job is usually interesting enough to keep me from getting bored.	JSMS
03.	It seems that my friends are more interested in their jobs.	JSMS
04.	I considered my job rather unpleasant.	JSMS
05.	I enjoy my work more than my leisure time.	JSMS
06.	I am often bored with my job.	JSMS
07.	I feel fairly well satisfied with my present job.	JSMS
08.	Most of the time I have to force myself to go to work.	JSMS
09.	I am satisfied with my job for the time being.	JSMS
10.	I feel that my job is no more interesting than others I could get.	JSMS
11.	I definitely dislike my work.	JSMS
12.	I feel that I am happier in my work than most other people.	JSMS
13.	Most days I am enthusiastic about my work.	JSMS
14.	Each day of work seems like it will never end.	JSMS
15.	I like my job better than the average worker does.	JSMS
16.	My job is pretty uninteresting.	JSMS
17.	I find real enjoyment in my work.	JSMS
18.	I am disappointed that I ever took this job.	JSMS

**Table A2:** Overview of Professional Aspects of Job Satisfaction.

SN	Questions Regarding Various Aspects of Respondents’ Present Job	Job Satisfaction Items	Response Patterns	Reference
01.	Are you satisfied with the salary/wages that you draw from your present job?	Pay/ salary/wages	Yes/No	QMSPA
02.	Are you satisfied with the promotional opportunity at your present job?	Promotion	Yes/No	QMSPA
03.	Are you satisfied with the job status at your present job?	Job status	Yes/No	QMSPA
04.	Are you satisfied with the job security at your present job?	Job security	Yes/No	QMSPA
05.	Are you satisfied with the working condition of your present job?	Working conditions	Yes/No	QMSPA
06.	Are you satisfied with the behaviour of your present boss?	Behavior of boss	Yes/No	QMSJF
07.	Are you satisfied with the opportunity for open communication with your present boss?	Open communication	Yes/No	QMSJF
08.	Are you satisfied with the autonomy in work at your present job?	Autonomy in work	Yes/No	QMSJF
09.	Are you satisfied with the recognition that is given for good work at your present job?	Recognition	Yes/No	QMSJF
10.	Are you satisfied with the opportunity of participation in decision making at your present job?	Participation	Yes/No	QMSJF
11.	Are you satisfied with the relation with colleagues at your present job?	Relation with colleagues	Yes/No	QMSJF
